# 14-3-3 Proteins Participate in Light Signaling through Association with PHYTOCHROME INTERACTING FACTORs

**DOI:** 10.3390/ijms151222801

**Published:** 2014-12-09

**Authors:** Eri Adams, Celine Diaz, Jong-Pil Hong, Ryoung Shin

**Affiliations:** 1 RIKEN Center for Sustainable Resource Science, 1-7-22 Suehiro-cho, Tsurumi-ku, Yokohama, Kanagawa 230-0045, Japan; E-Mails: eri.adams@riken.jp (E.A.); celine.diaz@cragenomica.es (C.D.); jongpil.hong@riken.jp (J-P.H.); 2 Center for Research in Agricultural Genomics (CRAG), Universitat Autònoma de Barcelona, Cerdanyola del Vallès, 08193 Barcelona, Spain

**Keywords:** PHYTOCHROME INTERACTING FACTOR (PIF), 14-3-3 protein, light signaling, photomorphogenesis, *Arabidopsis thaliana*, plant, protein-protein interaction

## Abstract

14-3-3 proteins are regulatory proteins found in all eukaryotes and are known to selectively interact with phosphorylated proteins to regulate physiological processes. Through an affinity purification screening, many light-related proteins were recovered as 14-3-3 candidate binding partners. Yeast two-hybrid analysis revealed that the 14-3-3 kappa isoform (14-3-3κ) could bind to PHYTOCHROME INTERACTING FACTOR3 (PIF3) and CONSTITUTIVE PHOTOMORPHOGENIC1 (COP1). Further analysis by *in vitro* pull-down assay confirmed the interaction between 14-3-3κ and PIF3. Interruption of putative phosphorylation sites on the 14-3-3 binding motifs of PIF3 was not sufficient to inhibit 14-3-3κ from binding or to disturb nuclear localization of PIF3. It was also indicated that 14-3-3κ could bind to other members of the PIF family, such as PIF1 and PIF6, but not to LONG HYPOCOTYL IN FAR-RED1 (HFR1). 14-3-3 mutants, as well as the PIF3 overexpressor, displayed longer hypocotyls, and a *pif3* mutant displayed shorter hypocotyls than the wild-type in red light, suggesting that 14-3-3 proteins are positive regulators of photomorphogenesis and function antagonistically with PIF3. Consequently, our results indicate that 14-3-3 proteins bind to PIFs and initiate photomorphogenesis in response to a light signal.

## 1. Introduction

Members of the 14-3-3 protein family bind to phosphorylated target proteins and regulate various physiological processes in all eukaryotes [1]. They are one of two phosphopeptide-binding proteins that exist in plants together with forkhead-associated (FHA) domain proteins and bind to the consensus amino acid sequences, RX_1-3_(pS/T)XP, where pS/T represents phosphorylated Ser/Thr [2]. The “classical” binding partners of 14-3-3 proteins involve metabolic enzymes, such as nitrate reductase (NR), sucrose-phosphate synthase (SPS) and glutamine synthetase (GS) [2,3]. 14-3-3 proteins are also known to interact with membrane proteins, including proton-pumps, ATP-binding cassette (ABC) transporters, cation channels and aquaporins [4]. Recent findings have highlighted the involvement of 14-3-3 proteins in the signaling of phytohormones, such as abscisic acid (ABA), brassinosteroids (BR), ethylene, cytokinins, auxin and gibberellins (GA) [4,5]. Many more 14-3-3 clients are being proposed [6]. In our previous work, a group of protein candidates that may interact with 14-3-3κ and χ isoforms *in vivo* in a model plant, *Arabidopsis thaliana*, have been identified through an affinity purification strategy combined with LC-MS/MS [7]. 14-3-3κ and 14-3-3χ are plant-specific non-ξ-14-3-3 isoforms [8], and they have been reported to be phosphorylated by a sucrose non-fermenting-related kinase, SnRK2.8 [9]. In addition to the proteins that are reported as being involved in plant nutrient metabolism [7], light-related proteins were suggested as binding partners of 14-3-3 proteins in this work. In plants, 14-3-3 proteins have been known to participate in light signaling. This notion is supported by the previous observation that 14-3-3 proteins interact with blue (B) light receptor kinases, PHOTOTROPIN1 (PHOT1) and PHOTOTROPIN1 (PHOT2), a central regulator of photoperiodic flowering, CONSTANTS (CO), and the downstream components, FLOWERING LOCUS T (FT) and TERMINAL FLOWER1 (TFL1) [10,11,12,13,14,15].

A basic helix-loop-helix (bHLH) transcription factor, PHYTOCHROME INTERACTING FACTOR3 (PIF3), is a key repressor of photomorphogenesis in the dark. PIF3 specifically binds to a *cis*-acting regulatory element, G-box, in the promoters of various light-regulated genes [16] to regulate their expression. Targets of PIF3 include *LATE ELONGATED HYPOCOTYL* (*LHY*) and *CIRCADIAN CLOCK ASSOCIATED1* (*CCA1*), the components required for a functional circadian clock [17]. PIF3 also negatively regulates light responses through interaction with HISTONE DEACETYLASE15 (HDA15) and synergistic transcriptional repression of genes related to chlorophyll biosynthesis and photosynthesis in the dark [18]. PIF3 is regulated at both the transcriptional and the post-translational levels. A noncoding RNA, *HIDDEN TREASURE* (*HD*), has recently been identified as repressing *PIF3* transcription by associating with the chromatin of the first intron of *PIF3* and promoting photomorphogenesis in continuous red (cR) light [19]. At the post-translational level, light rapidly induces the conversion of red/far-red (R/FR) light photoreceptors, phytochrome A (phyA) and phyB, to their active forms and consequent nuclear import and promotes direct interaction with DNA-bound PIF3 [20,21,22]. It has been reported that nuclear transport of phyB is mainly facilitated by PIF1, PIF3, PIF4 and PIF5, whereas that of phyA is facilitated by FAR-RED ELONGATED HYPOCOTYL1 (FHY1) and FHY1-LIKE (FHL) [23,24,25]. Upon PIF3-phy interaction in nuclear speckles, PIF3 is phosphorylated, and both PIF3 and phyB are ubiquitinated by Bric-a-Brack/Tramtrack/Broad (BTB)-Cullin3-type E3 ligases, LIGHT-RESPONSE BTBs (LRBs) and degraded by the 26S proteasome [26,27,28]. It has been shown recently that the *N*-terminal half of phyB is capable of inhibiting PIF3 from binding to its target DNA, but is not sufficient for speckle localization, PIF3 degradation nor inhibition of skotomorphogenesis [29,30]. In parallel with LRB-dependent degradation, PIF3 promotes interaction between phyB and another E3 ligase, CONSTITUTIVE PHOTOMORPHOGENIC1 (COP1), which is a negative regulator of photomorphogenesis, and COP1 ubiquitinates phyB (not PIF3), which is thereby degraded by the 26S proteasome [31]. By contrast, PIF3 degradation by R/FR light requires phyA, phyB and phyD, not COP1, but PIF3 accumulation in the dark requires COP1 [32]. Light-induced phosphorylation of PIF3 is crucial for its rapid degradation and consequent initiation of photomorphogenesis. A recent study has indicated that multiple Ser/Thr phosphorylation events in response to light collectively promote PIF3 degradation [33].

Our previous work has reported the involvement of 14-3-3κ and χ isoforms in plant nutrient metabolic pathways [7]. From the screening conducted [7], some light-related proteins, including PIF3, were also recovered. Although a proteomic analysis has revealed that PIF4 co-purifies with the 14-3-3ω isoform [6], direct interaction between 14-3-3 proteins and PIFs has not been confirmed. In this work, the role of 14-3-3 proteins in the light signaling pathway is discussed. Yeast two-hybrid and *in vitro* pull-down assays indicated the interaction between a 14-3-3 protein and light-related proteins, such as PIF3 and COP1. Mutants of 14-3-3s, as well as a PIF3 overexpressor showed longer hypocotyl lengths compared to the wild-type in cR light, suggesting that 14-3-3 proteins are important for the functional regulation of photomorphogenesis, likely the result of the interaction with phosphorylated PIF3 and the phyB-PIF3-COP1 signalosome.

## 2. Results

### 2.1. Yeast Two-Hybrid Analysis of 14-3-3 with Light-Related Proteins

In our previous report, we identified 14-3-3 interacting partners through an affinity purification method using His-tagged 14-3-3-coated beads and elution by adding 14-3-3 binding phosphopeptides followed by sequencing through trypsin digestion and mass spectrometry. 14-3-3 proteins have been shown to bind to nitrogen- and sulfur-related proteins and play roles in nutrient metabolic pathways in *Arabidopsis* [7]. Proteins involved in potassium and phosphate transport have also been suggested as the binding partners of 14-3-3 proteins. From the same affinity purification screening, we found a series of light-related proteins that might interact with 14-3-3 proteins (Table 1). PHOT2 and phyB are B and R/FR light photoreceptors, respectively [34,35], and COP1 and PIF3 are known to interact with phyB [20,31]. PHOT2 is also reported to bind with COP1 [36]. RGA1 and RGL2 are members of DELLA proteins and reported to bind to PIF3 [37].

**Table 1:** List of light-related 14-3-3 candidate partners recovered from the affinity purification strategy [7]. The *Arabidopsis* Genome Identification (AGI) and presence of the 14-3-3 binding motif in each protein are also indicated.

AGI	Gene Name	14-3-3 Binding Motif
AT2G18790	PHYTOCHROME B (PHYB)	yes
AT5G58140	PHOTOTROPIN2 (PHOT2)	yes
AT2G30520	ROOT PHOTOTROPISM2 (RPT2)	incomplete
AT2G32950	CONSTITUTIVE PHOTOMORPHOGENIC1 (COP1)	yes
AT5G42970	CONSTITUTIVE PHOTOMORPHOGENIC8 (COP8)	incomplete
AT1G09530	PHYTOCHROME INTERACTING FACTOR 3 (PIF3)	yes
AT2G01570	REPRESSOR OF GA1-3 1 (RGA1)	yes
AT3G03450	RGA-LIKE2 (RGL2)	incomplete

**Figure 1:**
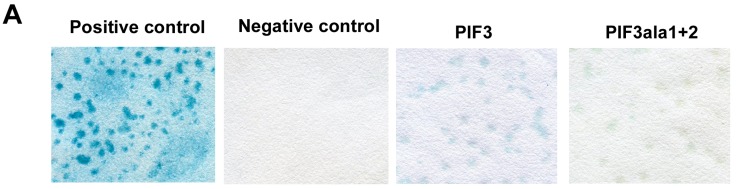

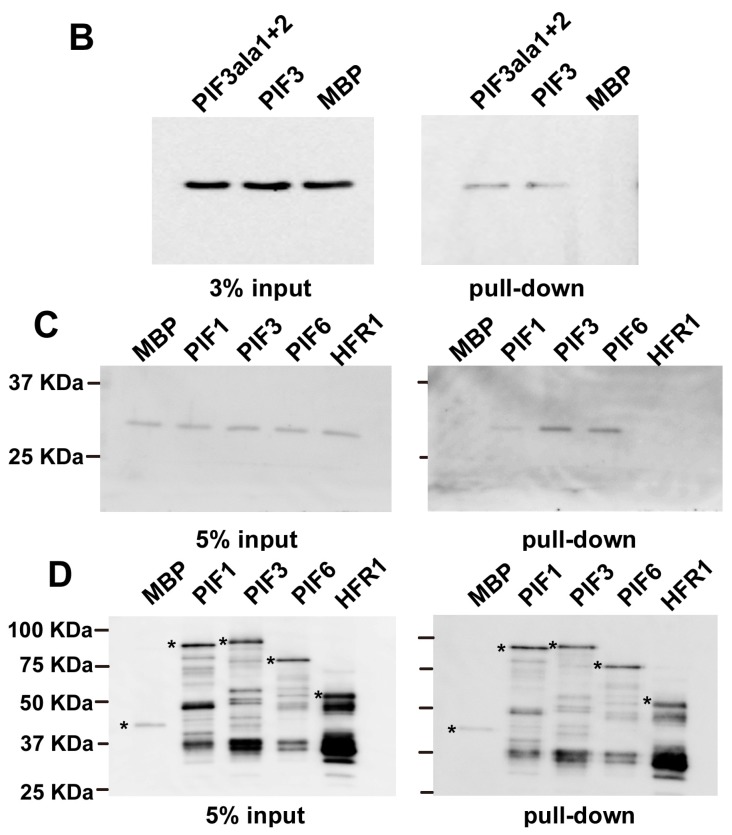
(**A**) Yeast two-hybrid analysis of 14-3-3κ with PIF3 and PIF3ala1+2. The interaction was determined by filter assay. A combination of pEXP32-Krev1 with pEXP22-RalGDS-wt was used as a positive control and pEXP32-Krev1 with pEXP22-RalGDS-m2 as a negative control; (**B**) Western blot analysis of HIS-14-3-3κ pulled down with maltose-binding protein (MBP, negative control), MBP-PIF3, MBP-PIF3ala1+2 bound to amylose resin beads and 3% of total input blotted with antiHis antibody; (**C**) Western blot analysis of His-14-3-3κ pulled down with MBP (negative control), MBP-PIFs bound to amylose resin beads and 5% of total input blotted with antiHis antibody; and (**D**) Western blot analysis of MBP-PIFs after pull-down and 5% of total input blotted with antiMBP antibody. Asterisks indicate the signal for each MBP-bound PIF protein. These experiments were repeated multiple times and representative data are shown.

Yeast two-hybrid analysis of PIF3 and COP1 with 14-3-3κ was performed. Since 14-3-3 proteins selectively bind to phosphorylated proteins, putative phosphorylation sites of PIF3, which are predicted to be important for 14-3-3 binding, were deactivated by converting Ser to Ala (PIF3ala1+2), and its binding capability to 14-3-3κ was assessed by the yeast two-hybrid assay. PIF3ala1 corresponds to Ser-23 exchanged with Ala (PIF3S23A), while PIF3ala2 corresponds to both Ser-115 and Ser-116 exchanged with Ala (PIF3S115AS116A, Figure S1). A typical blue color, due to β-galactosidase activity, was visible for PIF3 and COP1 (Figure 1A and Figure S2). PIF3ala1+2 also showed a faint, but visible blue signal (compared to the negative control), suggesting that 14-3-3κ likely interacts with PIF3, COP1 and PIF3ala1+2 in the yeast system.

### 2.2. In Vitro Pull-Down of 14-3-3 with PIFs

To confirm the interaction between 14-3-3κ and PIF3, *in vitro* pull-down was performed. His-tagged 14-3-3κ was successfully pulled down with maltose-binding protein (MBP)-tagged PIF3 and MBP-PIF3ala1+2, but not with the MBP negative control (Figure 1B), suggesting that 14-3-3κ binds to PIF3 and that the disturbance of these phosphorylation sites does not interrupt this interaction, being consistent with the yeast two-hybrid results. Other members of the PIF family were also investigated for their ability to bind with 14-3-3 proteins. PIF1 and PIF6 were shown to interact with 14-3-3κ, but not LONG HYPOCOTYL IN FAR-RED1 (HFR1), according to the *in vitro* pull-down assay (Figure 1C,D).

### 2.3. Subcellular Localization of GFP-PIF3ala1+2

**Figure 2:**
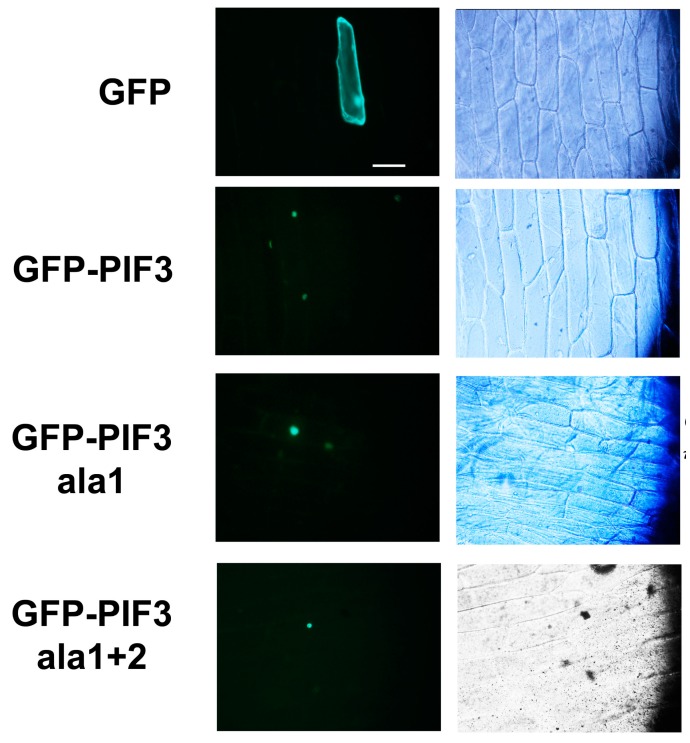
Subcellular localization of GFP, GFP-PIF3, GFP-PIF3ala1 and GFP-PIF3ala1+2. Fluorescence (**left**) and light field (**right**) images of onion epidermis cells bombarded with corresponding DNA are shown. The scale bar indicates 100 μm.

The light-related transcription factor PIF3 is known to localize in the nucleus, where it binds to phyB in response to light [32]. In order to investigate the integrity of PIF3 with deactivated putative phosphorylation sites, GFP-fused *Arabidopsis* PIF3 constructs were created, and subcellular localization of each protein was analyzed in onion cells through transient expression. In contrast to the GFP control, whose signal was distributed throughout the cytoplasm, GFP-PIF3 showed clear localization in the nuclei (Figure 2). This nuclear localization was maintained for GFP-PIF3ala1 and GFP-PIF3ala1+2.

### 2.4. Light Phenotype of 14-3-3 Mutants

**Figure 3:**
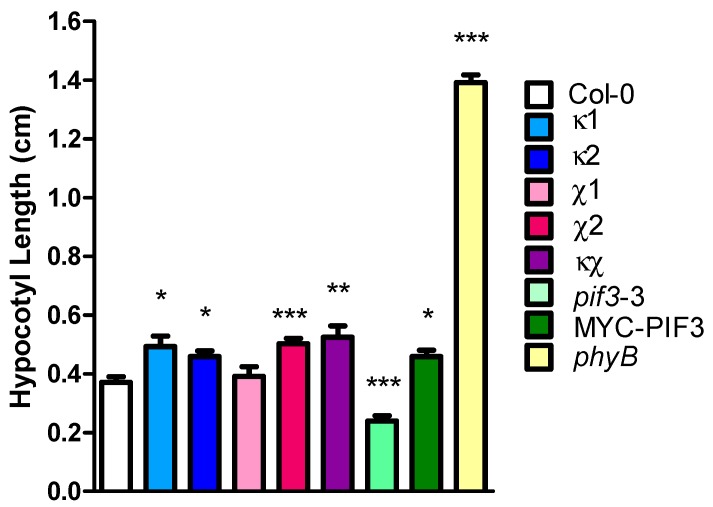
Hypocotyl lengths of 14-3-3 mutants grown in continuous red (cR) light. Col-0 (wild-type), κ1 (salk_148929), κ2 (salk_001375), χ1 (salk_150150), χ2 (salk_142285), κχ double-mutant (salk_001375; salk_142285), *pif3*-3, MYC-PIF3 and *phyB* were germinated and grown in cR light for five days. Statistically significant differences compared to the wild-type are indicated with asterisks (*n* > 11): * *p <* 0.05, ** *p <* 0.01, *** *p <* 0.001. These experiments were repeated multiple times, and representative data are shown.

An interaction between 14-3-3κ and light-related proteins, such as PIF3 and COP1, was demonstrated. Since 14-3-3 proteins regulate various physiological processes by binding with phosphopeptides in all eukaryotes [1], the role of 14-3-3 proteins in light signaling was investigated. Hypocotyl elongation in plants is inhibited in the light as a part of the photomorphogenic response. 14-3-3 homozygous knockout mutants [7], including κ1 (salk_148929), κ2 (salk_001375), χ1 (salk_150150), χ2 (salk_142285), κχ double-mutant (salk_001375; salk_142285), as well as *pif3*-3 mutant and MYC-PIF3 overexpressor were grown in cR light, and hypocotyl lengths were compared to those of Col-0 (wild-type) and an R light photoreceptor mutant, *phyB*. All of the 14-3-3 mutants, except χ1, displayed longer hypocotyls compared to Col-0 in cR light (Figure 3), indicating that 14-3-3 proteins are important for R light perception and response in *Arabidopsis*. By contrast, overexpression of *PIF3* caused longer hypocotyls, and a mutation in *PIF3* caused shorter hypocotyls compared to Col-0 in cR light (Figure 3). This is consistent with the role of PIF3 as a negative regulator of photomorphogenesis. These phenotypical observations reinforce the role of 14-3-3 proteins in light signaling.

## 3. Discussion

PIF3 is a bHLH transcription factor, which serves as a negative regulator of photomorphogenesis. Since perception of and response to light signals are of the utmost importance in plants, the mechanisms by which PIF3 is regulated have been intensively studied. Recent findings have filled some gaps in our knowledge, and what appears to be the whole picture of this mechanism has been revealed. That is, PIF3 binds to the promoter of the light-related genes and regulates their transcription in the dark [16]. Upon light signaling, phytochromes are activated and translocated to the nuclear speckle, where they bind to PIF3 [20,21,22]. PIF3 is phosphorylated, ubiquitinated by E3 ligases, called LRBs, and degraded by the 26S proteasome, so that the light-related processes can be induced [26,27,28]. Here, we propose 14-3-3 proteins serving as yet another layer of the regulatory components of PIF-dependent light signaling in plants.

According to our yeast two-hybrid and *in vitro* pull-down results, it is suggested that 14-3-3κ directly binds to PIF3, as well as to PIF1 and PIF6. Due to their extremely short half-life in light [38], phosphorylated forms of PIFs were not detected *in vivo*. Thus, further confirmation of an interaction between 14-3-3κ and PIFs by an *in vivo* co-immunoprecipitation assay has not been successful so far. Although PIF3 is considered to be the predominant repressor of photomorphogenesis, other PIFs, including PIF1 and PIF6, are known to bind with phytochromes in response to light and participate in the repression of photomorphogenesis in the dark [38,39,40,41]. It is therefore assumed that 14-3-3κ is a general regulatory component of light signaling through the interaction with PIFs. By contrast, our data suggest that 14-3-3κ does not bind with HFR1, an atypical bHLH protein. Although HFR1 shares sequence similarity with PIF3, it has an atypical basic region and cannot bind to phytochromes [42]. Recent findings have indicated that HFR1, together with PIF3-LIKE1 (PIL1), function antagonistically to PIFs and promote photomorphogenesis [43]. These results, together with our results, suggest that 14-3-3 proteins specifically associate with PIFs to regulate a photomorphogenic response.

Since 14-3-3 proteins selectively bind to phosphorylated proteins, putative phosphorylation sites on the 14-3-3 binding motifs of PIF3 were disrupted by exchanging Ser with Ala (PIF3ala1+2). However, PIF3ala1+2 protein was capable of proper nuclear localization and 14-3-3 binding. PIF3ala1 (Ser-23 exchanged with Ala) and PIF3ala2 (each of Ser-115 and Ser-116 exchanged with Ala) regions are the only sites on PIF3 that harbor the complete 14-3-3 binding motif, RXXSXP. According to a previous report, the phosphopeptide signals of S23 and S116 are approximately 20% in both dark and R light [33]. These findings suggest that incomplete motifs, such as RXXS/T, may be the actual 14-3-3 binding sites of PIF3 (Figure S1), as is the case for PHOT1 [11]. It has recently been reported that multiple light-induced phosphorylation sites in PIF3 are collectively required for rapid degradation of PIF3 and phyB [33]. It is speculated that ala1+2 was not sufficient to cancel PIF3’s function or that other phosphorylation sites are important for its localization and 14-3-3 binding.

Our phenotypical data show that 14-3-3 mutants have longer hypocotyls in cR light, suggesting their reduced sensitivity to R light. This phenotype is similar to that of the PIF3 overexpressor and opposite to that of the *pif3* mutant, consistent with the previously reported data [44]. From these results, the involvement of 14-3-3κ in light signaling and the antagonistic role to that of PIF3 can be inferred. The reduced sensitivity of 14-3-3 mutants is not as much as that of *phyB*, since the *phyB* mutant shows much longer hypocotyls in cR light. It is possible that members of the 14-3-3 family function redundantly or that the participation of other components is significant in light signaling.

**Figure 4:**
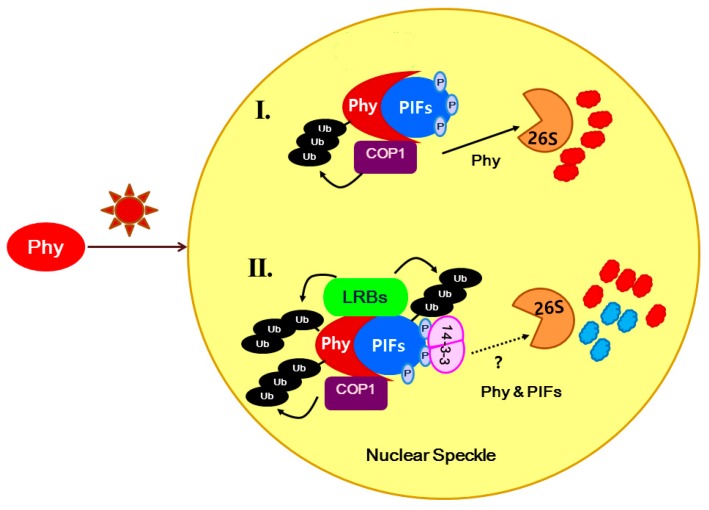
Model for the mode of action of 14-3-3κ in the context of light signaling. **I.** Upon light illumination, phytochromes (Phy) rapidly convert to an active form and translocate into a nuclear speckle where they bind to negative regulators of light signalling, PHYTOCHROME INTERACTING FACTORs (PIFs). An E3 ligase, CONSTITUTIVE PHOTOMORPHOGENIC1 (COP1) polyubiquitinates Phy, but not PIFs, to be degraded by the 26S proteasome, serving as a negative feedback mechanism in light signaling; **II.** Phy-bound PIFs are phosphorylated, E3 ligases, LIGHT-RESPONSE BTBs (LRBs), polyubiquitinate both Phy and PIFs to be degraded by the 26S proteasome, and consequently photomorphogenesis is initiated. 14-3-3κ is predicted to bind with phosphorylated-PIFs and either escort LRBs to the Phy-PIF complex or stabilize the LRB-Phy-PIF complex to promote PIFs degradation as positive regulators of light signalling.

The summary of our findings, together with the previous findings, is depicted in Figure 4. In response to light signaling, 14-3-3κ is predicted to bind to phosphorylated-PIFs or possibly bridge PIFs-COP1, according to our yeast two-hybrid analysis. It can be speculated that 14-3-3κ either escorts LRBs to the Phy-PIF complex or stabilizes the LRB-Phy-PIF complex, so that PIFs are degraded by the 26S proteasome and photomorphogenesis initiates, serving as positive regulators of light signaling. The role of 14-3-3 proteins in protein degradation through ubiquitin E3 protein ligases has been reported in *Arabidopsis*. A stress-responsive F-box protein, FBS1, has been shown to interact with 14-3-3 proteins, and this interaction is important for FBS1 degradation by the 26S proteasome [45]. 14-3-3 proteins also interact with components of an E3 ligase, ETO1/ELOs, and promote their degradation in ethylene biosynthesis [46], suggesting that regulation of protein degradation by 14-3-3 proteins may be a general mechanism in plants. The involvement of 14-3-3 proteins in light signaling can be elaborated further by investigation of the other light-related proteins identified in our analysis detailed above. The list of candidate proteins recovered from affinity purification analysis contains photoreceptors phyB and PHOT2 and DELLA proteins, such as RGA1 apart from PIF3 and COP1. PHOT2 has been reported to interact with the 14-3-3λ isoform [12], which has strong sequence similarity to 14-3-3κ. The interaction of 14-3-3 proteins with phyB could be indirect through the PIF-COP1 complex. RGA1 is involved in GA signaling and known to interact with PIF3 to inhibit its transcriptional activity [37]. Hence, 14-3-3 proteins might regulate GA light signaling through direct or indirect binding to RGA1 or the PIF3-RGA1 complex, respectively. Taken together, the data presented here indicate that 14-3-3 proteins bind to PIFs and initiate photomorphogenesis in response to light signaling, possibly through PIF degradation.

## 4. Experimental Section

### 4.1. Plant Material and Growth Conditions

The *Arabidopsis thaliana* accession Col-0, *pif3*-3 [47],* phyB* (salk_069700), 14-3-3κ mutants (salk_148929 and salk_001375), 14-3-3χ mutants (salk_150150 and salk_142285) and the double-mutant 14-3-3κχ (salk_001375; salk_142285) [7] were used. MYC-PIF3 overexpressor was created using pGW21 [36]. The full-length PIF3 coding sequence was cloned with gene-specific primers with the 5' TOPO recognition site; forward (f): ACACCATGCCTCTGTTTGAGC and reverse (r): TCACGACGATCCACAAAACTGATCAG. The amplified fragments were cloned into pENTR using the Gateway^®^ system (Invitrogen, Carlsbad, CA, USA). The sequence was confirmed, and the cDNA fragments were recombined into the destination vectors through the Gateway^®^ LR Clonase^®^ reaction (Invitrogen). *Agrobacterium*-mediated transformation of Col-0 was performed, and transformants were selected for their resistance to antibiotics. Seeds were surface-sterilized with 70% (*v*/*v*) ethanol and 0.05% (*v*/*v*) Triton X-100 and sown on media containing half MS salt (Duchefa Biochemie, Haarlem, The Netherlands), pH 5.8, with NaOH and 0.6% phytoagar (Duchefa Biochemie). After stratification at 4 °C, plants were placed in a vertical orientation in a growth chamber at 22 °C in continuous red light with a light intensity of 10 µmol/m^2^/s. The hypocotyl lengths of 5-day-old seedlings were measured using ImageJ (http://rsbweb.nih.gov/ij/, National Institute of Health, Bethesda, MD, USA). One-way ANOVA with Dunnett’s multiple comparison posttest (*p* < 0.05) was performed using Prism (GraphPad Software, La Jolla, CA, USA) to determine the statistical significance.

### 4.2. Yeast Two-Hybrid 

pDEST22-PIF3, pDEST22-COP1 and pDEST32-14-3-3κ were created. The MaV203 yeast strain was transformed with each DNA and plated on selective media according to the manufacturer’s instruction (Invitrogen). pEXP32-Krev1 with pEXP22-RalGDS-wt and pEXP22-RalGDS-m2 was used as a positive and a negative control, respectively (Invitrogen). For the mutated versions of PIF3, pENTR-PIF3 was mutated using GeneTailor Site-Directed Mutagenesis System (Thermo Fisher Scientific, Waltham, MA, USA) and the primer sets: PIF3ala1f: CTGCTCAAGACAGGAACCCTGCTCCACCTGTAG, PIF3ala1r: AGGGTTCCTGTCTTGAGCAGATTCAAGCTTAG, PIF3ala2f: CTGATTTCTTGCGTGATGTGGCGGCTCCTGTTACTGTC and PIF3ala2r: CACATCACGCAAGAAATCAGAGCAATATCCATC. The filter assay was performed to analyze the interaction between bait and prey proteins, according to the Clontech Yeast Protocols Handbook (Takara, Shiga, Japan).

### 4.3. In Vitro Pull-Down

MBP-tagged PIFs were created using the pMAL system (New England BioLabs, Ipswich, MA, USA). Primers used for cloning are PIF1f: CGGAATTCATGCATCATTTTGTCCCTGCA, PIF1r: GCCTGCAGTTAACCTGTTGTGTGGTTTCC, PIF3f: CACCGAATTCATGCCTCTGTTTGAGCTTTT, PIF3r: GTCGACTCACGACGATCCACAAAACTG, PIF6f: CGGAATTCATGATGTTCTTACCAACCGAT, PIF6r: GCCTGCAGTCATCTGTTAGTTTTCCTTGA, HFR1f: CGGAATTCATGTCGAATAATCAAGCTTTC and HFR1r: GCCTGCAGTCATAGTCTTCTCATCGCATG. The PIF3 coding sequence with the enzyme sites was amplified by Phusion High-Fidelity DNA Polymerase (New England BioLabs), cloned into pENTR (Invitrogen), and the sequence was confirmed. The PIF3 fragment and the pMAL vector were cut by the restriction enzymes, *Eco*RI and *Sal*I (Takara), and recombined by T4 DNA ligase (Takara). For PIF1, PIF6 and HFR1, the coding sequences with the enzyme sites were amplified by Ex Taq DNA polymerase (Takara), ligated into pGem-T Easy vector (Promega, Fitchburg, WI, USA), and the sequence was confirmed. The genes in the pGEM-T Easy vector and the pMAL vector were cut by the restriction enzymes, *Eco*RI and *Pst*I (Takara), and recombined by T4 DNA ligase (Takara). pMAL-PIFs were transformed into *Escherichia coli* BL21 (DE3), induced by 0.3–0.5 mM isoprophyl β-d-1-thiogalactopyranoside (IPTG) at 25–28 °C for 5–6 h and purified using amylose resin according to the manufacturer’s instructions (New England BioLabs). The details of the His-tagged 14-3-3 proteins were described previously [9]. For the pull-down assay, 0.3–20 µg of 14-3-3κ protein were incubated with equivalent or half the amount of MBP or 5–10-times more MBP-PIFs in the MBP column buffer at 4 °C in dark on a rotator for 5–6 h, followed by incubation with amylose resin (New England BioLabs) overnight. The sample mixture was washed and eluted with elution buffer containing 100 µM MG132 and 10 mM maltose. Retained proteins were resolved by SDS-PAGE and detected using anti-MBP antibody (New England BioLabs) and anti-His antibody (Covance, Princeton, NJ, USA).

### 4.4. Subcellular Localization of PIF3 in Onion

GFP-PIF3 was created using pGW6 [36], as mentioned in Section 4.1. Purified gold particles were coated with GFP-PIF3 DNA and bombarded into a freshly prepared onion (*Allium cepa*) epidermis layer using Biolistic Particle Delivery System PDS-1000/He (Bio-Rad, Hercules, CA, USA). Onion cells were incubated at 22 °C in the dark overnight and observed under a fluorescence microscope, BX51 (Olympus, Tokyo, Japan).

## Supplementary Materials

Supplementary figures can be found at http://www.mdpi.com/1422-0067/15/12/22801/s1.
